# Production and characterization of collagen films containing essential fatty acids for cicatrization

**DOI:** 10.1186/1753-6561-8-S4-P14

**Published:** 2014-10-01

**Authors:** Fernando Mendonça Diz, Catharina Grace Santos, Andriele Mendonça Barbosa, Klebson Silva Santos, Lucas Reis d' Costa, Isabelle Souza de Mélo Silva, Clauberto Rodrigues de Oliveira, Juliana Cordeiro Cardoso, Ricardo Luiz Cavalcanti de Albuquerque Júnior, Francine Ferreira Padilha

**Affiliations:** 1 Universidade Tiradentes, Aracaju, Brazil; 2 Universidade Federal de Sergipe, RENORBIO, Aracaju, Brazil

## Background

The skin is the largest organ of the human body and corresponds to the first line of defense against external aggression. The extent and the commitment of organ and tissue will depend on the severity and intensity of trauma. The regeneration and healing are two processes that compose the restoration of tissues. It is a complex process involving a number of cellular and molecular events, as well as biochemical and physiological phenomena. The process of healing by secondary intention occurs in tissue repair in wounds with a higher degree of injury (injuries that may involve the subcutaneous tissue, muscle or bone). A range of biomaterials have been used in an attempt to incorporate substances that facilitate the process of tissue repair, especially the collagen to be biocompatible and not immunogenic. Essential fatty acids (EFAs) has been used in various types of injuries and different stages of the healing process. Therefore, this study aims to develop, characterize and physically evaluate the effect of collagen films containing EFAs on wound healing of second intention.

The cicatricial part has already generated paper in Acta Cirurgica Brasileira, vol.28 no.5 São Paulo May 2013 (http://dx.doi.org/10.1590/S0102-86502013000500005).

## Methods

The films were produced by the method of casting, where two formulations were prepared using collagen hydrolyzate more commercial EFAs (EFA 50 and 100). The films were characterized by mechanical analysis, macroscopic permeability of water vapor and optical microscopy. For the study of healing surgical wounds were made on the dorsum of 80 rats, covered with films of Collagen (FCOL), collagen films over 50 EFA (FEFA50), and collagen films with EFA100 (FEFA100). Wounds without toppings were used as control (control group). The animals (rats) were euthanized at 3, 7,14, and 21 days. Macroscopic indices of wound contraction (IWC) were obtained and performed histological analysis of the scar area using conventional microscopy and polarized light.

## Results and conclusion

The FEFA50 and FEFA100 films formed transparent, continuous, homogeneous and easy to use. With the addition of EFA50 EFA100 and movies, these had the thickest, stretching, tension and lower Young's modulus. In biological assay (scarring) not macroscopic signs of abscesses or hypertrophic scarring in either group were observed. At 7 days, the IWC observed in FEFA50 was higher compared to the control group, while at 14 days, and FEFA50 FEFA100 these indices showed increased compared to control and movies only COL. FEFA50 FEFA100 and promoted increased neutrophilic infiltration in 3 days, but the lifocítico infiltrate was reduced to 14 days. It was also observed the early development of granulation tissue, re-epithelialization of dermal annexes and better collagen organization. This study suggests that films of collagen as an additive containing the essential fatty acids, particularly FEFA50, are promising for use as roofing or wound dressings for dermal enhancement of scar repair process biomaterials.
